# Release of tree species diversity follows loss of elephants from evergreen tropical forests

**DOI:** 10.1098/rspb.2024.2026

**Published:** 2025-04-02

**Authors:** John Terborgh, Lisa Ong, Lisa Clare Davenport, Wei Harn Tan, Alicia Solana Mena, Kim McConkey, Ahimsa Campos-Arceiz

**Affiliations:** ^1^ Department of Biology, University of Florida, Gainesville, FL, USA; ^2^ Tropical Environments and Societies, James Cook University Division of Tropical Environments and Societies, Cairns, Queensland, Australia; ^3^ Southeast Asia Biodiversity Research Institute & Center for Integrative Conservation, Megafauna Ecology and Conservation Group, Xishuangbanna Tropical Botanical Garden, Menglun, People’s Republic of China; ^4^ Chinese Academy of Sciences, Beijing, Yunnan, People’s Republic of China; ^5^ The University of Nottingham Malaysia, Semenyih, Selangor, Malaysia; ^6^ School of Environmental and Geographical Sciences, The University of Nottingham Semenyih, Selangor, Malaysia, Selengor, Malaysia

**Keywords:** elephants, forests, herbivory, tree species diversity

## Abstract

We report on a decade of research on elephant impacts in equatorial evergreen forests in Gabon and Malaysia, comparing sites with (+) and without (−) elephants and documenting major differences in forest structure, tree species composition and tree species diversity. In both regions, we compared sites supporting natural densities of elephants with otherwise undisturbed sites from which elephants had been absent for several decades. Elephant (+) sites supported low densities of seedlings and saplings relative to elephant (−) sites. In Lope National Park, Gabon, 88% of saplings and small trees (<20 cm dbh) were of species avoided by elephants, implicating forest elephants as powerful filters in tree recruitment. In Malaysia, Asian elephants showed strong preferences for monocots over dicots, as we found through both indirect and direct means. Loss of elephants from both Asian and African forests releases diversity from top-down pressure, as preferred forage species increase in abundance, leading to increased density of small stems and tree species diversity. In contrast, loss of other major functional groups of animals, including top carnivores, seed predators and seed dispersers, often results in negative impacts on tree diversity.

## 1. Background

Herbivory is a top-down force on plants, just as predation is a top-down force on animals, and like predation, herbivory is regulated in nature by strategies to limit or prevent it, such as chemical and physical defences, low detectability, refuging in inaccessible sites, etc. [1–3]. Megaherbivores present a challenge to this general rule in that megaherbivores are not limited by predators, so possess the potential to overwhelm vegetation [4,5]. Elephants and other large herbivores, including rhinoceroses, hippos, giraffes and even unregulated deer, have the potential to modify habitats, in extreme cases flipping them to alternative states [6–8].

Here we inquire into the impacts of megaherbivores, principally elephants, on closed-canopy tropical forests, an environment that has received relatively little attention in the herbivory literature [9]. Most of the foliage in such a forest is displayed 20 or more metres above the ground, limiting the ability of earthbound megaherbivores to transform the vegetation. Importantly, the presence of herbivores capable of breaking, uprooting, stripping or otherwise destroying saplings presents a challenge to tree recruitment [2]. Nevertheless, herbivores do not destroy tall forests because they exercise selectivity in the choice of foraging targets and avoid some species. Selectivity could be mediated by palatability and nutritional value offset by chemical or physical deterrents [10,11].

The preferred method for studying herbivore impacts is via exclosure experiments [12], but at the present writing, we know of no reports of such experiments in humid tropical forests containing elephants and other large herbivores. Exclosure experiments conducted in savannah and woodland environments with contemporary megafauna have yielded consistent results across sites and herbivore species. Herbivore exclusion leads to increased density of woody stems, especially palatable species, and reduction of avoided species [13,14].

Foraging conditions in humid tropical forests differ from those in more open environments. Forest productivity is concentrated in the canopy, and canopy trees are too large to be pushed over, even by elephants. Accessible forage is confined to the understorey, where light is limited, productivity is low, leaf lifetimes are long and growth is slow. Elephants and other megafauna are able to harvest some of the productivity of the canopy by consuming fallen fruit [15]. But fruit is a seasonal resource and available in limited quantities when in season, so forest-dwelling elephants must forage for foliage throughout the year.

Successful tree recruitment is severely constrained in a forest with browsing megafauna because light-limited saplings can require years or even decades to grow through a window of vulnerability from a diameter of approximately 0.1 to approximately 3−4 cm [16,17]. Escape from the risk of being foraged can thus occur only under restricted circumstances. Saplings of fast-growing, light-demanding species can potentially attain escape diameter in just a year or two in the largest naturally occurring gaps [18]. However, the saplings of most species found in the tropical forest understorey are shade-tolerant and limited to slow growth and a prolonged period of exposure to browsing.

## 2. Experimental design and site characteristics

Here we review the results of a decade of research in forests supporting ecological densities of African forest elephants (*Loxodonta cyclotis*) in Gabon and Asian elephants (*Elephas maximus*) in Malaysia. We compared forest structure, composition and diversity between sites in Gabon and Malaysia supporting ecological densities of elephants (elephant (+) sites) and sites from which elephants had been driven out or removed several decades previously (elephant (–) sites). We infer elephant impacts in tall equatorial forests from both indirect and direct observations and show that these impacts are similar in kind to those described for savannah elephants [14] and to those of lesser herbivores in situations of high herbivore abundance: deer [19,20], moose [21], beaver [22] and even leaf-cutter ants [23].

All sampled sites were located between 5.5° N and 2° S in tall evergreen forest. In Gabon, we measured forest structure and composition in 1 ha plots at 11 sites between 2009 and 2011. All plots were located within protected areas supporting a range of elephant densities. In addition, we sampled stems <10 cm dbh in 21 m^2^ × 100 m^2^ strips at six sites, four of which were in protected areas (details in [24,25]). We highlight results from Lope National Park as it contains one of the highest recorded elephant densities anywhere (≥1 per km^2^) [26].

In Gabon, the elephant (−) site was the Foret de la Mondah, a protected forest 20 km north of Libreville, the capital. The forest in Lope National Park had not been exploited by humans, at least in modern times, whereas the Forêt de la Mondah showed patchy evidence of prior slash-and-burn activity in the presence of okoumé trees (*Aucoumea kleiniana*), a long-lived early successional species. In selecting sampling sites, we avoided areas with okoumé trees [24]. According to local informants, elephants had not been seen in the Mondah forest since the 1980s.

In Malaysia, vegetation sampling took place between 2015 and 2019 and was limited to stems <10 cm dbh at seven sites in elephant (+) Royal Belum State Park and seven sites in the elephant (−) Krau Wildlife Reserve (details in [27]). Elephant densities in Royal Belum were low, roughly estimated at 0.1 per km^2^ [28]. Our sampling sites in both the Belum park and the Krau reserve were in unlogged lowland dipterocarp forest. The last wild elephants in Krau were captured and translocated to larger forest reserves in the early 1990s [27,28].

## 3. Synthesis of results

### (a) Forest structure

Lewis *et al*. [29] give mean figures for per-hectare densities of trees ≥10 cm dbh from plots in Central Africa (425), Asia (Borneo; 602) and central/east Amazonia (597). The values for Central Africa and Amazonia are close to what we reported from just four plots in these two regions: Gabon (sites supporting high elephant densities; mean 377) and Peru (no megafauna; mean 618) [25]. Moreover, the size distribution of trees in Africa is distinct. There are roughly three times as many trees ≥60 cm dbh in Gabonese as in Peruvian forests per 1000 stems ≥10 cm dbh, and a pronounced paucity of small trees <20 cm dbh [25,29]. Small trees (≥10 and <20 cm dbh) predominate in Malaysian forests over larger trees by a ratio of nearly 2 to 1 in the 50 ha plots at Pasoh (68%) and Lambir (63%) versus 62% in the Peruvian Amazon (mean of 11 sites) versus 54% in Gabon [27,30].

The number of small stems <1 m tall and saplings (≥1 m tall and <10 cm dbh) were lower at Lope (+) than at Mondah (−) (table 1). Our findings were similar in Malaysia, where the density of tree saplings was substantially greater at elephant (−) Krau Wildlife Reserve (Krau) than at elephant (+) Royal Belum State Park (Belum; table 1).

**Table 1.** Seedling, sapling and tree densities in Gabon, Malaysia and Peru. The hypothesis being tested here is that there is no difference between elephant (+) and elephant (−) sites. The Peru data are for reference only. Seedlings and saplings sampled in 100 m^2^ strips (*n* = 5 in Gabon and Peru; *n* = 7 in Malaysia). Trees sampled in standard 1 ha plots (trees not sampled in Malaysia). The original data are contained in [24,25,27].

location	all seedlings	tree seedlings	small tree saplings	large tree saplings	trees
	<1 m tall per m^2^	≥10 cm, <1 m tall per m^2^	≥1 m, <1 cm diameter per 100 m^2^	≥1 cm, <10 cm diameter per 100 m^2^	≥10 cm diameter per ha
Gabon					
Lope (+)	3.25 ± 2.54***	0.75 ± 0.90***	10.6 ± 6.7**	26.8 ± 5.9	397
Mondah (−)	15.5 ± 7.3	2.8 ± 2.7	44.4 ± 10.6	42.4 ± 15.8	415
Malaysia					
Belum (+)	23.6 ± 13.3^*^	2.54 ± 5.7	49 ± 10.3***	37 ± 7.1^*^	—
Krau (−)	48.4 ± 31.0	4.70 ± 5.6	108 ± 10.1	59 ± 15.6	—
Peru					
Madre de Dios (−)	21.4 ± 16.1	2.3 ± 1.8	69.3 ± 8.7	39.0 ± 7.6	600 ± 10.7^a^

*p < 0.05, **p < 0.01,***p < 0.001 by nonparametric Mann–Whitney test.

^a^ Six upland sites representing diverse soil types in Madre de Dios, Peru.

### (b) Forensics of megafaunal foraging: stem breaks

One of the most frequent and visible manifestations of megaherbivore foraging is the presence of scars on saplings marking the height of past breaks [31]. Such scars offer the best available species-level indirect evidence of megafaunal foraging. From the height distribution of break scars, one can surmise the species identity of foragers [32,33]. Breaks can also be caused by falling debris and by crown dieback during drought or light starvation [34]. Foraging breaks can be crudely distinguished by subtracting ‘background’ breaks tallied in forests lacking megaherbivores from the observed height distribution of breaks in forests with megaherbivores [24,27].

**Figure 1.**
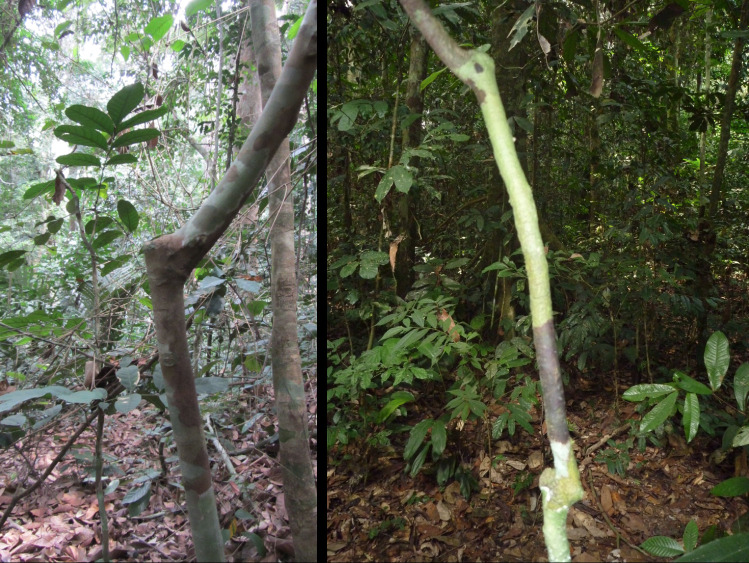
Saplings in Lope National Park, Gabon, showing scars of one (left) and two (right) former breaks.

Break scars are frequent on saplings in the 1−5 cm diameter class in Gabon, averaging 105 breaks per 100 stems in three forests supporting unpoached elephant populations. The incidence of breaks exceeds the number of stems because some stems carry two, three or even four scars at successively higher levels (figure 1). For reference, the saplings in a forest in the Peruvian Amazon lacking megafauna carried 34 scars per 100 stems distributed over a wide range of heights. Subtracting the Amazonian non-foraging breaks from the breaks recorded in Gabon yielded a unimodal height distribution with a peak at 1.1−2.0 m and reduced numbers of breaks at ≤1.0 m and ≥ 2.1 m [24]. In Malaysia’s Belum (+) forest, the incidence of breaks was less than in Gabon at 70 per 100 stems and unexpectedly deficient in breaks in the 1.0−2.0 m height range. Instead, more than half (51%) of all breaks were at <1.0 m, implying that pigs, not elephants, were responsible for most of them [35].

Break scars provide only half a picture; the other half is invisible and consists of the stems that are uprooted during foraging or die after being broken. To determine whether the impact of megafaunal foraging on sapling survival is heavy or light, we conducted an experiment in the Royal Belum State Park (Malaysia) that consisted of cutting 1228 saplings at heights characteristic of foraging pigs (*Sus scrofa*; 0.5 m), Asian tapirs (*Tapirus indicus*; 1.0 m) and Asian elephants (1.5 m) [35]. After 13 months, we found that 89% of the cut saplings were alive, 7% were confirmed dead, and 4% were not found and could have been alive or dead. Of the saplings cut at 0.5 and 1.0 m, 66% carried previous break scars at a mean height of 0.4 m. The low height implicated pigs as the principal instigator of break scars at Belum (+) [33]. A discrete signal for elephants was not discernible from background noise, although elephants could not be ruled out as causing some of the 25% of breaks in the height range of 1.1–2.0 m [27].

### (c) Selectivity of foraging: indirect evidence

The selectivity of megafaunal foraging is important because it has the potential to influence forest species composition and species diversity. Broad patterns of selectivity can be inferred from indirect evidence of various kinds [11]. An opportunity to investigate foraging selectivity in Gabon appeared fortuitously in the form of an article on elephant foraging damage to saplings and small trees in Lope National Park [36]. Assessed levels of damage allowed Cardoso *et al*. [36] to distinguish species that are ‘strongly preferred’ (*n* = 9), ‘somewhat preferred’ (*n* = 11) and ‘avoided’ (*n* = 9) by foraging elephants. The classification allowed us to use data we had collected at Lope several years earlier to test the null hypothesis that species of saplings and small trees <20 cm dbh were neutral with respect to elephant foraging preference (table 2). The observed frequencies of the three preference categories departed radically from the expected frequencies, such that avoided species comprised 88% of all saplings and small trees in the understorey (table 2). As the lists published in [36] included only a minority of the species found in interior forest at Lope, the results in table 2 must be considered approximate.

**Table 2.** Expected versus observed occurrence of species of saplings and small trees at Lope, in Gabon, in relation to elephant foraging preference under the assumption of equal likelihood of occurrence in forest interior stands. The expected occurrence is based on the preference status of 29 tree species given in [36].

	small saplings ≥1 m tall, ≤1 cm dbh		large saplings ≥1 cm, <10 cm dbh		trees ≥10 cm, <20 cm dbh	
preference status	exp.	obs.	exp.	obs.	exp.	obs.
strongly preferred	5.6	1	12.7	2	29.5	2
somewhat preferred	6.8	6	15.6	5	36.0	4
avoided	5.6	11	12.7	34	29.5	91
chi-square	*p* = 0.082		*p* < 0.0001		*p* < 0.0001	

However, corroboration of the result can be found in the composition of a 1 ha tree plot at Angak in Lope National Park, which contained 47 species of trees among 397 stems ≥10 cm dbh. Four species made up 53% of the total (*Centroplacus glaucinus* 9%, *Diospyros dendo* 26%, *Diospyros soyauxii* 6%, *Greenwayodendron suaveolens* 12%; Y. Malhi 2010, unpublished data). For such a small number of species to make up more than half the stand in an equatorial evergreen forest is highly unusual. All the species listed are understorey trees that only infrequently grow into the ≥20 cm diameter class, suggesting that they are shade tolerant and slow growing and therefore must pass slowly through the window of vulnerability to elephant foraging. High abundance could imply that they are disfavoured by foraging elephants.

Support for this hypothesis is contained in substantial increases in the proportion contributed by these four species to successive size classes: small saplings, 0.30, large saplings, 0.46 and small trees, 0.77. Correspondingly, the proportions of all other species combined show the opposite trend: 0.7, 0.54 and 0.23, respectively. The picture that emerges from these findings is that avoided species are greatly overrepresented in African elephant (+) forests and that preferred species are heavily suppressed.

In Malaysia, evidence of foraging selectivity pertained to both vegetation structure and composition. Stem numbers of small and large saplings were 2.2 and 1.6 times greater at Krau (−) than at Belum (+), and palms >1 m tall were more than 6 times greater at Krau (table 1) [27]. Tellingly, the Krau (−) forest abounded in monocots, including palms, rattans, bamboo and pandans [27]. In contrast, the forest at Lope, in Gabon, was essentially lacking in palms and other woody monocots, leaving African forest elephants to forage exclusively on dicot saplings.

### (d) Selectivity of foraging: direct evidence

Thanks to the generous collaboration of Malaysia’s Kuala Gandah National Elephant Conservation Center, we were able to observe the foraging of unrestrained wild-caught Asian elephants that had been brought into captivity. We arranged for the animals to be released by their mahouts within the Krau preserve in areas supporting two habitats (lowland dipterocarp forest or early second growth). Elephants free to forage at will were observed for one-half-hour sessions (*n* = 40). The units of observation were trunkfuls delivered to the mouth of an elephant. Mouthfuls were observed at close range (<5 m), and the type of plant being consumed was recorded. In addition, at the conclusion of a foraging session, the area foraged was searched for broken stems and other evidence of foraging. Nearby areas with equivalent vegetation were surveyed as controls. Full details are reported in [37].

Elephants foraging in natural forests consumed a wide range of plant materials, including (as % of mouthfuls) bamboo (7.0), grasses (0.0), herbs (4.1), palms (39.4), lianas (19.9) and saplings of dicot trees (29.6). Elephants were highly selective of monocots, especially palms, for which the preference ratio (plants consumed relative to availability) was 5.4, contrasting with 0.4 for dicot tree saplings. Of the latter, 40% were <1 cm dbh and 60% >1 cm dbh. Elephants are destructive foragers and broke many stems they did not consume. Of the tree saplings foraged (108 stems), 25% were uprooted, 35% broken, 20% stripped of leaves, 3% debarked and 17% suffered branch removal. Within foraged areas, 68% of stems sustained serious damage, including 66% of palms, 79% of lianas and 62% of tree saplings. In contrast, in the early successional environment where light is abundant, fast-growing large tree saplings were positively selected (preference ratio 1.6), and the rate of stem damage doubled. Overall, tree saplings were broken at a mean height of 1.1 m and a diameter of 1.7 cm [37].

### (e) Species diversity

The role of megafauna in regulating the species diversity of tropical forests has, to our knowledge, not been previously investigated, although it has been well documented that elephants can reduce the species diversity of savannah woodlands by imposing high mortality on preferred species [12,38]. In both Gabon and Malaysia, we were able to compare plant diversity in elephant (+) and elephant (−) forests [24,27]. In both cases, the diversity of small and large saplings (Fisher’s alpha) was substantially greater (by factors of 2.1–3.8) in forests lacking elephants (table 3) [24,27].

**Table 3.** Diversity (Fisher’s alpha ± s.d.) of small and large saplings at upland megaherbivore (+) and (−) sites in Gabon and Malaysia. Saplings sampled in 100 m^2^ strips (*n* = 5 at Lope and Mondah and *n* = 7 at Belum and Krau). Data for trees ≥10 cm dbh refer to standard 1 ha tree plots at Lope and Mondah. We did not install tree plots in Malaysia. Statistical tests refer to elephant (+) versus elephant (−) sites. The original data are contained in [24,27].

locality	saplings ≥1 m tall, <1 cm dbh	saplings ≥1 cm dbh, <10 cm dbh	trees ≥10 cm dbh, <20 cm dbh	trees ≥20 cm dbh
Africa				
Lope (+)	9.2 ± 7.0	5.2 ± 1.9	7.4	16.0
Mondah (−)	19.7 ± 8.8	15.9 ± 5.2**	22.0	16.2
Malaysia				
Belum (+)	28.1 ± 7.7	26.6 ± 18.4	—	—
Krau (−)	91.4 ± 30.1^*^	99.8 ± 41.1**	—	—

*p < 0.05, **p < 0.01

Saplings: two-tailed Mann–Whitney test for Lope versus Mondah and Belum versus Krau.

Note that the diversity of canopy trees (≥20 cm dbh) at Lope is twice that of the small tree class (≥10 cm, <20 cm dbh), whereas the small tree class is more diverse at Mondah. Given that every stem that reaches the canopy must pass through the understorey, higher diversity in the canopy might seem counterintuitive. We pursued this further using data from 10 Gabon tree plots from sites supporting a range of elephant densities from low to very high [25]. Fisher’s alpha for small trees (≥10 cm, <20 cm dbh) is 19.5 ± 6.5 (mean ± s.d.) and for trees ≥20 cm dbh is 23.5 ± 8.3 (Wilcoxon signed-rank, paired, two-tailed, *p* = 0.047). Contrasting with this are data from six upland tree plots in Madre de Dios, Peru. Mean Fisher’s alpha for small trees (≥10 cm, < 20 cm dbh) was 74.2 ± 15.3, but only 60.6 ± 15.9 for trees >20 cm dbh (Wilcoxon signed-rank, paired, two-tailed, *p* ≤ 0.0001).

### (f) Diversity release

Diversity in all stem size classes smaller than trees ≥20 cm dbh was substantially greater at Mondah (−) than at Lope (+), though not all contrasts reached the *p* < 0.05 level (table 3). Large tree diversity at Lope and Mondah was equal, suggesting that the signal of elephant extirpation had not yet reached the canopy at Mondah. Qualitatively similar results were obtained in Malaysia, where Fisher’s alpha for both small and large saplings was much higher (91 and 100) at Krau (−) than at Belum (+): 28 and 27, respectively [27].

## 4. Discussion

### (a) Forest structure

Elephants are well-known to act as ‘ecological engineers’ by imposing strong top–down effects on the structure of savannah and woodland vegetation in those portions of Africa and southern/southeastern Asia, where large herbivores still persist [12,39–41]. Both African savannah and Asian elephants engage in selective foraging, a process that holds the potential for modifying the species composition of habitats in comparison with megafauna-free control sites [5,11,41–43].

**Figure 2.**
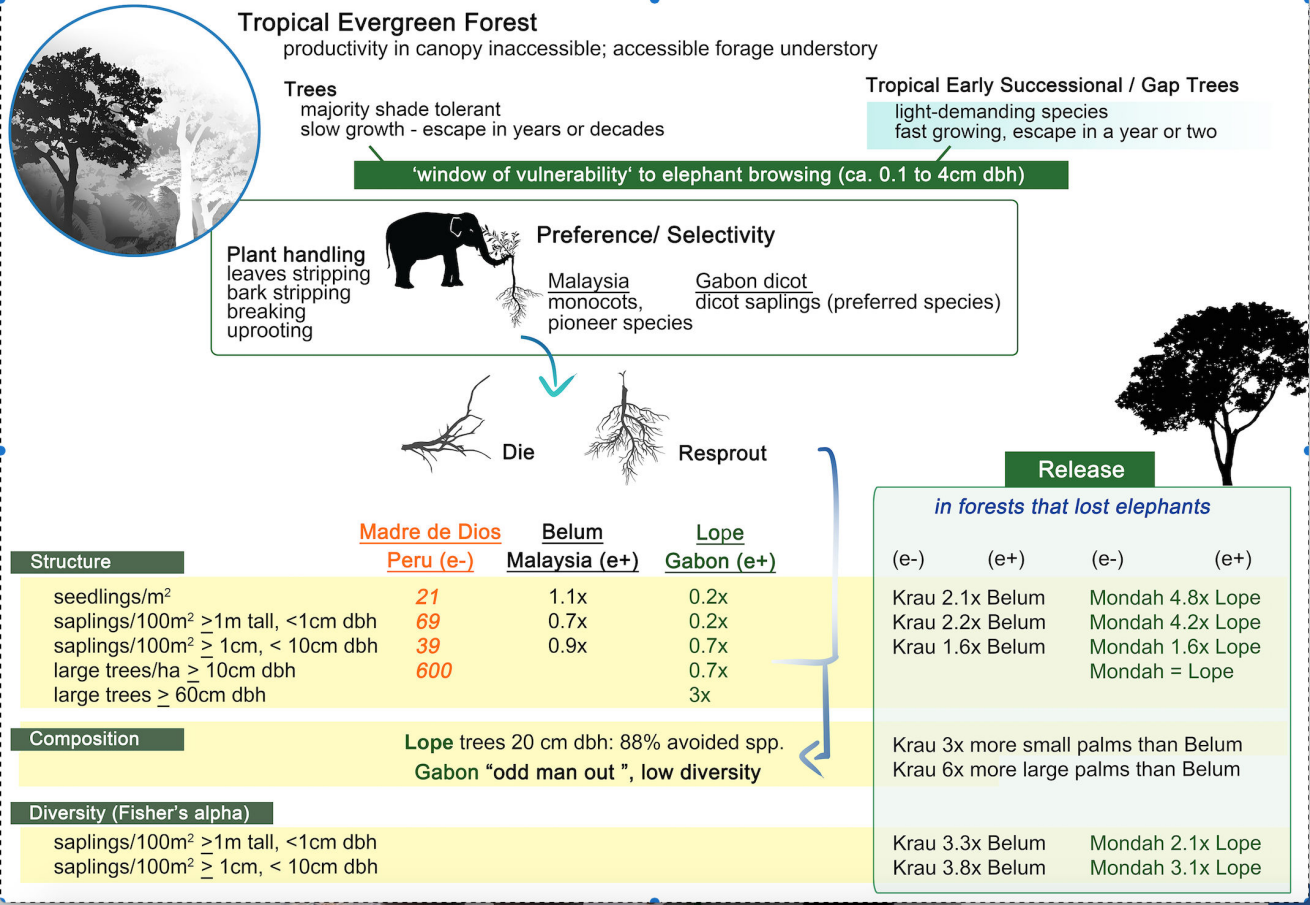
Here we summarize our results comparing elephant impacts in Gabon versus Malaysia: foraging selectivity at the top, structural impacts in yellow and diversity effects in green.

Comparisons of elephant (+) and elephant (−) forests in Gabon and Malaysia yielded similar results (figure 2). Stem counts of small (≥1 m tall, <1 cm dbh) and large (≥1 cm, <10 cm dbh) saplings were markedly lower in elephant (+) forests, as also noted for Gabon [44]. In both Gabon and Malaysia, there are smaller herbivores that forage on small saplings, among them, duikers (*Cephalophini* spp.) in Africa and wild boar, sambar (*Rusa unicolor*), muntjac (*Muntiacus muntjac*) and tapir in Malaysia. Stems ≥1 cm dbh are the main targets of elephants, as confirmed using stem breaks and through direct observations in Malaysia. Most stems broken by elephants in both Gabon and Malaysia were in the 1−4 cm range. Stems attaining ≥5 cm dbh have successfully passed through the window of vulnerability. We did not detect a signal of elephant foraging on dicot saplings in Malaysia, but the density of elephants in the Belum (+) park is roughly a 10th (estimated at roughly 0.1 per km^2^) of that at Lope in Gabon. Evidence of stem breakage by pigs, however, was strong at both Belum (+) and Krau (−) [35].

Gabonese (+) forests contained many more large trees (≥60 cm dbh) and many fewer small trees (≥10 cm, <20 cm dbh) than forests in the Peruvian Amazon [25]. These contrasts were most pronounced at Lope, a park with one of the highest recorded elephant densities at ≥1.0 per km^2^ [26]. Thus, both the number and size distribution of woody stems seem to be strongly altered by elephant foraging in closed-canopy African evergreen forests. We did not install 1 ha tree plots in Malaysia, so do not have comparable structural data for Belum and Krau. However, we did show, using 100 m^2^ strip samples, that palms were far more abundant at Krau (−) than at Belum (+), indicating that the impacts of Asian elephants on the structure and composition of Malaysian forests differ from those of African forest elephants, which forage primarily on dicots.

Berzaghi *et al*. [45] present a model from which they conclude that the high frequency of very large trees and relative scarcity of small trees in Central African forests are attributable to elephant foraging. In particular, they infer that the paucity of small trees (≤20 cm dbh) in these forests reduces underground competition and increases understorey light availability, allowing trees reaching the canopy to attain greater size. We regard this as a hypothesis in need of testing. Berzaghi *et al*. [11] estimate that forest structural changes due to elephant loss could result in a 6–9% reduction in aboveground carbon stocks in Central African forests.

### (b) Foraging selectivity

There are parallels between our Gabon and Malaysia results as well as differences. African forest elephants exclusively foraged on dicot saplings, whereas Asian elephants showed a strong preference for monocots. Our finding that 88% of saplings and small trees in the forest at Lope in Gabon are of avoided species indicates that elephant foraging is a powerful filter on species composition that must strongly regulate successful recruitment of saplings into the canopy.

Distinct foraging preferences reflect differences between the two elephant species in both dentition and habitat. African elephants (*Loxodonta* spp.) possess lophodont dentition, associated with browsing, whereas Asian elephants (*E. maximus*) possess hypsodont dentition, associated with grazing [46]. Forests are the main habitat of the African forest elephant, but Asian elephants are scarce in uniformly forested habitats, such as in Belum, and more common in open habitats such as savannahs and grasslands. Differences in dentition and habitat preference are likely to underlie the order of magnitude difference in the abundance of elephants at Lope and Belum. The overall impact of Asian elephants in forested habitats is consequently less than that of African forest elephants.

### (c) Species diversity

Tree species diversity in African evergreen forests is notoriously low, prompting Richards [47] to declare Africa as the ‘odd man out’ among the tropical forest regions of the world [25]. Some investigators (e.g. [48,49]) have ascribed low tree diversity to Africa’s climatic history, a history that includes strong evidence of droughts and fire. Without denying the possibility of climate history as a contributing factor, there are at least three biotic factors that contribute to Africa’s low hectare-scale tree diversity. First, the number of trees in African forests (approx. 400 per ha) is a third less than in Asian or American forests (approx. 600 per ha), decisively reducing per-ha species counts [25,29]. Second, elephant foraging filters out many palatable species at the sapling stage, lowering species diversity locally and leaving mostly a few unpalatable species to dominate the smaller stem size classes, a result also observed in forests overbrowsed by deer in North America [50]. Third, the species accumulation curve for African forests rises more slowly than in other tropical forests, implying more rare species. Among 13 tropical 50 ha plots, species numbers of African plots ranked fourth, fifth and sixth behind only the hyperdiverse plots at Lambir, Yasuní and Pasoh and ahead of plots in Colombia, Sri Lanka and Panama [30]. Thus, at the scale of 50 ha, Africa is not the odd man out.

Another anomalous feature of African forests is the rarity of saplings of canopy species. Many canopy species, including some of the most common, did not appear among the more than 5500 saplings documented in our samples [24]. Since all trees that attain canopy status must grow up through the understorey, one would logically suppose that small trees (≥10 cm, <20 cm dbh) should be more diverse than large trees (≥20 cm dbh), as we found in 6 Peruvian forests in which Fisher’s alpha for small trees (74) was greater than for large trees (61). Nevertheless, Gabonese forests showed the opposite trend, in that trees ≥20 cm dbh were more diverse than trees <20 cm dbh (Fisher’s alpha = 23.5 versus 19.5). Recruitment of many canopy species in the African forest is a nearly invisible process that must occur only infrequently and then perhaps in spatial or temporal refugia [2,18].

### (d) Diversity release

Selective herbivory can interact with interspecific competition to regulate plant species diversity either upwards or downwards [10,20]. In situations of high productivity that lead to intense plant-to-plant competition, herbivory can reduce competition by opening colonization sites, resulting in increased diversity in a manner analogous to the role of the starfish (*Pisaster*) in Paine’s famous experiment [13,20,51,52]. Herbivory in a forest understorey, however, can have the opposite effect of lowering diversity, in what might be termed a reverse Paine effect. Herbivore-mediated loss of plant diversity has been shown in long-sustained exclosure experiments in North America [50,53] and Panama [54]. Photosynthesis is strongly light-limited in a forest understorey, muting competition relative to open sites. Densities of small stems <1 m tall are low (approx. 20 per m^2^) in tropical forests in America, Africa and Malaysia [24,27,55]. At 20 stems per m^2^, competition between stems in the ground layer is much less than the asymmetric competition between small stems and trees in the canopy [56–58]. Accordingly, selective herbivory by white-tailed deer (*Odocoileus virginiana*) on ground-layer herbs in North American temperate forests, by leaf-cutter ants (*Atta* spp.) in Venezuela, and by elephants on saplings in tropical forests consistently reduces plant species diversity [7,23,53].

When top-down suppression of diversity is released through herbivore loss or exclusion, diversity increases, an effect apparent in both Gabon and Malaysia at sites from which elephants had been extirpated or removed 3 or 4 decades earlier. In contrast, long-established exclosure experiments in regions supporting hyperabundant densities of white-tailed deer in temperate North America have seen little recovery of ground-layer herbs on time scales of 5–28 years [20,59–61]. Diversity recovery could not occur unless seed sources remained in the neighbourhood, suggesting that, in tropical forests, even strongly preferred forage species persist in low numbers in natural refugia [2,3]. Suppressed recovery in temperate forests has been attributed to the smothering of seedling establishment by *Dennstaedtia punctilobula*, a browsing-resistant fern, a paucity of local seed sources, and the fact that many ground-layer herbs are dispersed by ants [53,61]. In Africa and Malaysia, species that disappear under heavy herbivore pressure appear to retreat into local scarcity or into habitats where they are less vulnerable but not global extinction [2,62,63]. Exceptions may occur under sustained high levels of herbivory, strong enough to flip the ecosystem into an alternative state [7]. As a phenomenon, diversity release is an important issue for conservation that merits further affirmation and quantification.

### (e) Ancien régime

Megafauna are realizing their last gasp in a world being overrun by humans [64]. The three surviving species of elephants are in decline, and the five species of rhinos are all in various stages of endangerment. We can study only fragments of what existed right up to the dawn of the human age. Prior to the catastrophic loss of megafauna over the last 50 000 years, large to giant herbivores abounded in all the ice-free continents, including many species of proboscidians. Ecologically, late Pleistocene proboscidians ranged from browsers (e.g. the North American mastodon (*Mammut americanum*) to grazers (e.g. the Columbian mammoth (*Mammuthus columbi*). Over large parts of the world, browsing and grazing proboscidians broadly coexisted: *Mammut* and *Mammuthus* in North America, *Stegodon* and *Elephas* in Asia, *Paleoloxodon* and *Mammuthus* in Europe, and *Elephas* and *Loxodonta* in Africa [65–67].

It is thus relevant to ask whether the megafaunal regimes we investigated in contemporary Gabon and Malaysia are representative of pre-overkill times. Evidence suggests that humans were not involved in driving megafaunal extinctions in Pleistocene Africa, and in any case, these extinctions were few in relation to those that occurred on other continents [68,69]. Data from Gabon reflect the pressure of herbivory exerted by the African forest elephant, a species specialized for the rainforest environment [70]. Our data from Gabon represent conditions as close to the pre-human as can still be found on the planet. Southeast Asia experienced more late-Pleistocene megafaunal extinctions, notably of *Stegodon*, which was a dedicated browser [65,68]. The remaining Asian proboscidean, *E. maximus*, is a mixed forager that reaches greatest abundance in savannah and woodland environments [4,66].

Historically, megafauna in Malaysia included *Stegodon* and both Javan (*Rhinoceros sondaicus*) and Sumatran (*Dicerorhinus sumatrensis*) rhinos, all forest-dwelling browsers (Javan rhinos might be mixed feeders, but they are currently restricted to forest habitats in Indonesia’s Ujung Kulon National Park). One can imagine that megafaunal impacts in the Malaysian forest would have been much greater prior to the extinction of *Stegodon* and the functional loss of the two rhinos [9]. Therefore, what we are able to report here may only hint at what might have been the condition of the Southeast Asian forest prior to megafaunal loss.

Perhaps our most notable finding is that the removal of elephants and other large herbivores from forests in Gabon and Malaysia results in a gain in local species diversity. In the two cases we were able to investigate, removal of elephants and perhaps other large herbivores, led to ‘release’ in stem numbers, structural parameters and, most importantly, species diversity. We do not want to imply that these changes are ‘good’; that would be inappropriate, because the changes are unnatural, and the ecosystem-level cascading effects of release are yet to be evaluated. In any case, contrary to many situations involving a massive intervention into the workings of nature, there appears to be little loss of species. The observed release of diversity could only occur if the additional plant species were present in the ecosystem in viable form, pointing to the conclusion that megaherbivores cause some species to be effectively invisible through rarity, but they do not cause their extinction. This leaves open the possibility that the system is inherently reversible, such that—following megaherbivore recovery—an ‘empty’ forest could revert to its former condition.

## Data Availability

Our manuscript is a review of previously published research and contains no new data, nevertheless, data from Gabon and Malaysia have been deposted in Dryad (https://doi.org/10.5061/dryad.1zcrjdg3n) [71].
